# Atlas of ticks (Acari: Argasidae, Ixodidae) in Germany: 1st data update

**DOI:** 10.1007/s10493-023-00784-5

**Published:** 2023-03-17

**Authors:** Franz Rubel, Stefan Zaenker, Alexander Weigand, Dieter Weber, Lidia Chitimia-Dobler, Olaf Kahl

**Affiliations:** 1 Unit for Veterinary Public Health and Epidemiology, University of Veterinary Medicine Vienna, Veterinärplatz 1, 1210 Vienna, Austria; 2 Hesse Federation for Cave and Karst Research, Fulda, Germany; 3 National Museum of Natural History Luxembourg, Luxembourg City, Luxembourg; 4 Fondation Faune-Flore, Musée National d’Histoire Naturelle, Luxembourg City, Luxembourg; 5 Bundeswehr Institute of Microbiology, Munich, Germany; 6 tick-radar GmbH, Berlin, Germany

**Keywords:** Tick map, Species distribution, Georeferenced data

## Abstract

The first data update of the atlas of ticks in Germany published in 2021 is presented here. This atlas provides maps based on georeferenced tick locations of 21 species endemic in Germany as well as three tick species that are regularly imported to Germany. The data update includes the following numbers of newly georeferenced tick locations: 17 *Argas reflexus*, 79 *Carios vespertilionis*, 2 *Dermacentor marginatus*, 43 *Dermacentor reticulatus*, 4 *Haemaphysalis concinna*, 3 *Haemaphysalis punctata*, 3 *Hyalomma rufipes*, 3 *Ixodes apronophorus*, 9 *Ixodes arboricola*, 1 *Ixodes ariadnae*, 30 *Ixodes canisuga*, 3 *Ixodes frontalis*, 80 *Ixodes hexagonus*, 3 *Ixodes lividus*, 497 *Ixodes ricinus/inopinatus*, 1 *Ixodes rugicollis*, 17 *Ixodes trianguliceps*, 14 *Ixodes vespertilionis*, and 45 *Rhipicephalus sanguineus* sensu lato. Old and new tick findings were mapped, such as the northernmost occurrence of *D. marginatus* in Germany observed in 2021, but also the historical records from the first descriptions of *I. apronophorus* and *I. arboricola*, which were georeferenced here for the first time. The digital dataset of tick locations available for Germany is supplemented by 854 new tick locations. These records increase the number of tick species mapped in the federal states Bavaria, Brandenburg and Mecklenburg Western Pomerania by five each, those in Berlin and Schleswig-Holstein by four each, those in Hamburg by three, those in Baden-Wuerttemberg, Bremen, Lower Saxony, Northrhine-Westphalia, Rhineland Palatinate and Thuringia by two each, and those in Hesse, Saxony and Saxony-Anhalt by one each. Thus, the first data update of the tick atlas in Germany and the underlying digital dataset significantly improve our knowledge of the distribution of these tick species and helps to investigate the effects of climate change and habitat changes on them.

**Supplementary Information:**

The online version contains supplementary material available at 10.1007/s10493-023-00784-5.

## Introduction

The atlas of ticks in Germany by Rubel et al. (2021) included 24 tick species, two species of Argasidae, namely *Argas reflexus* and *Carios vespertilionis*, and 22 species of Ixodidae. The latter include the endemic species *Dermacentor marginatus*, *Dermacentor reticulatus*, *Haemaphysalis concinna*, *Haemaphysalis punctata*, *Ixodes acuminatus*, *Ixodes apronophorus*, *Ixodes arboricola*, *Ixodes ariadnae*, *Ixodes canisuga*, *Ixodes frontalis*, *Ixodes hexagonus*, *Ixodes inopinatus*, *Ixodes lividus*, *Ixodes ricinus*, *Ixodes rugicollis*, *Ixodes simplex*, *Ixodes trianguliceps*, *Ixodes uriae*, and *Ixodes vespertilionis*. *Hyalomma marginatum* and *Hyalomma rufipes* each spring imported by migratory birds from the south as well as *Rhipicephalus sanguineus* sensu lato occasionally imported by dogs coming from the Mediterranean or other southern countries were shown in geographical maps.

Knowledge gaps in Rubel et al. (2021) were due to the fact that some relevant papers on ticks have not been written in English and, moreover, old articles and former journals are often not available in digital form. Much of the references used here can therefore not be found through common database queries, but only through expert knowledge. In addition, the lockdown in 2020/2021 caused by the COVID-19 pandemic made it more difficult to obtain literature from libraries that restricted their services or were completely closed (Nicola et al. 2020). Therefore, for example, the locations of the short-legged bat tick *C. vespertilionis* (Rupp et al. 2004) could only be assigned to the federal state of Bavaria. Due to the work of Sándor et al. (2021) geographical coordinates are now available. Some colleagues reacted to the atlas of ticks in Germany by pointing out the occurrence of ticks that were not taken into account by Rubel et al. (2021) by making their own studies accessible (Henkel et al. 1983). Last but not least, the historical work of Paul Schulze was included in the tick atlas. For example, the finding of *I. apronophorus* from the first description by Schulze (1924) and several findings of *I. arboricola* from the first description by Schulze and Schlottke (1929) have been georeferenced. Data from the publications mentioned, numerous other previously unavailable data sources and new tick findings of the authors might justify this data update, with which further gaps in the mapped tick occurrence in Germany have been closed. For this purpose, the new georeferenced locations and the updated distribution maps are presented here.

## Data and methods

The data used here are georeferenced tick locations in Germany described by Rubel et al. (2014, 2021) supplemented by 854 new records. The geographical coordinates of the new tick locations are provided in the supplement together with an indication of their accuracy and the sources. The coordinates are given in decimal degrees with a measure of accuracy identical to those previously introduced by Rubel et al. (2014, 2018, 2021).

The tick locations are mapped using R, a language and environment for statistical computing (R Development Core Team 2019). Artificial data clusters caused by single studies were reduced using a random selection and a thinning algorithm (Aiello-Lammens et al. 2019). For example, the newly georeferenced tick locations of the study by Centurier et al. (1979) and Hoffmann (1981) significantly increased the number of *R. sanguineus* sensu lato reports. However, only 42 out of 76 known locations were mapped to avoid overlapping location points.

Tick species, for which only a few locations are known, are grouped according to their host preferences as proposed by Hornok et al. (2020). For example, the bat ticks *C. vespertilionis*, *I. ariadnae*, *I. simplex*, and *I. vespertilionis* are shown in the same map.

## Results

The outcomes of this study are updated geographical maps that depict the occurrence of all tick species that have so far been reported in Germany. It should be noted that the widespread *I. inopinatus* (Hauck et al. 2019) has been combined with *I. ricinus* and they are called *I. ricinus/inopinatus* hereinafter as in Rubel et al. (2021). The improvements resulting from the data update are summarized in Table 1. Accordingly, the first data update increases the number of tick species mapped in the federal states Bavaria, Brandenburg and Mecklenburg Western Pomerania by five each, those in Berlin and Schleswig-Holstein by four each, those in Hamburg by three, those in Baden-Wuerttemberg, Bremen, Lower Saxony, Northrhine-Westphalia, Rhineland Palatinate and Thuringia by two each, and those in Hesse, Saxony and Saxony-Anhalt by one each. All tick species are presented below with a brief summary of the numbers of updated locations compiled for this study. If the ticks were collected from hosts, these are also mentioned. For information on the global distribution, biology, hosts, as well as the medical and veterinary importance of the tick species identified in this paper the reader is referred to Petney et al. (2012, 2015) and Rubel et al. (2014, 2021).

**Table 1:**
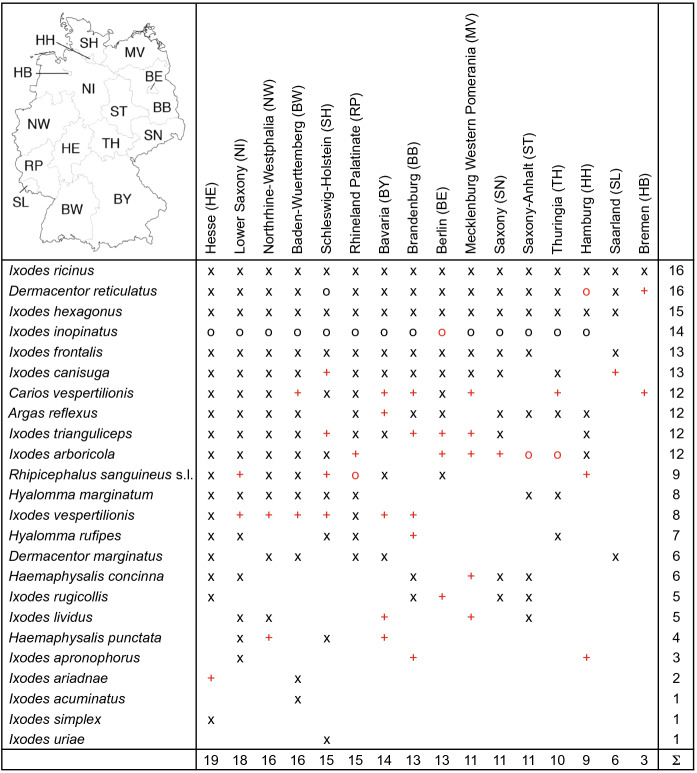
Occurrence of 24 tick species (Acari: Argasidae, Ixodidae) in the 16 German federal states: (x) georeferenced locations already mentioned in Rubel et al. (2021), (+) locations of the 1st data update, and (o) documented at the level of federal states (new data in red). (Colour figure online)

### *Argas (Argas) reflexus* (Fabricius)

**Fig. 1:**
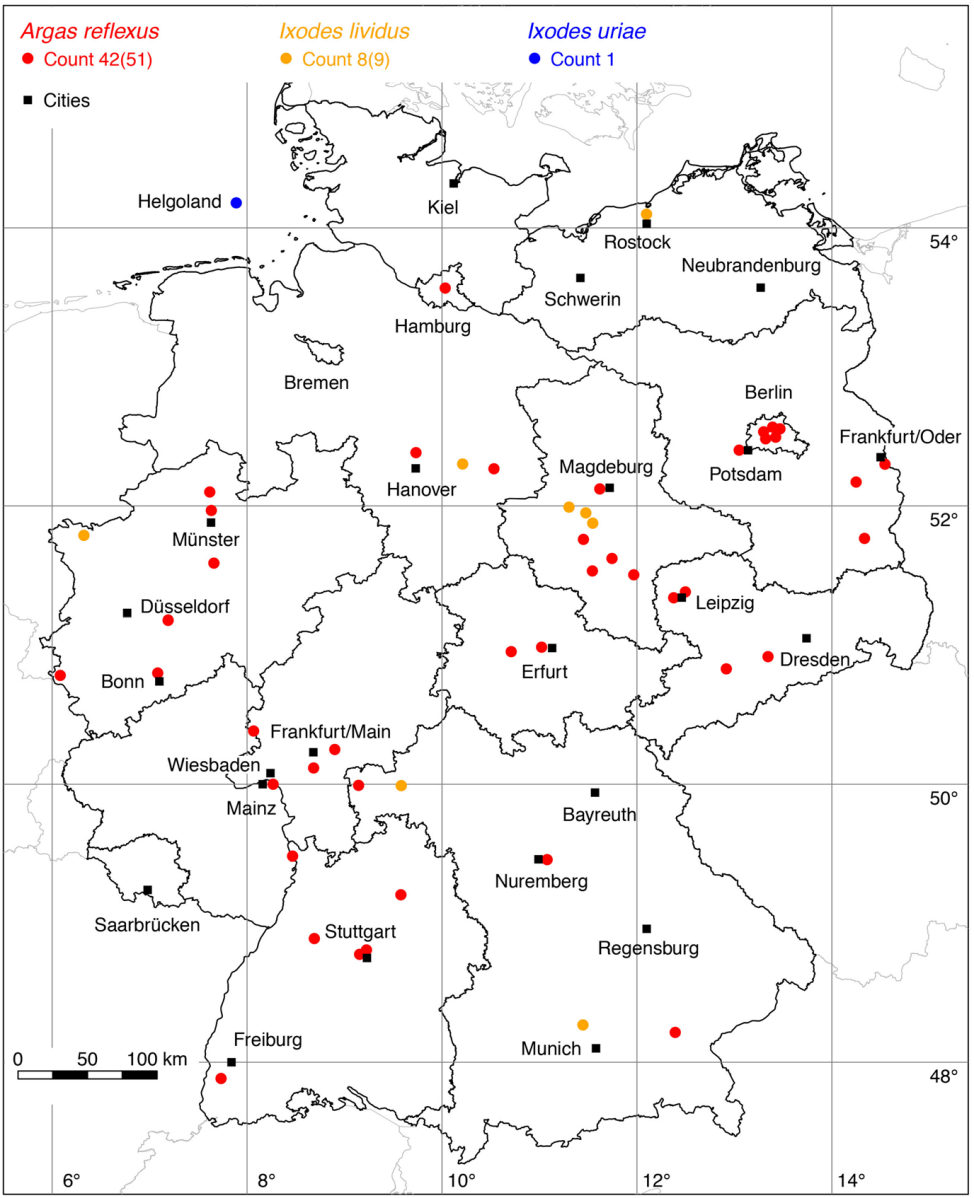
Recorded locations of *Argas reflexus*, *Ixodes lividus* and *Ixodes uriae* in Germany. (Color figure online)

The following 17 locations were added to the distribution map of the pigeon tick *A. reflexus*: 5 (Stadler and Schenkel 1940), 2 (Hoogstraal and Kohls 1960), 8 (Dautel et al. 1999), 1 (Rupp et al. 2004), 1 (Hoffmeister et al. 2008). A total of 42 out of 51 known *A. reflexus* observations is depicted in Fig. 1.

### *Carios (Carios) vespertilionis* (Latreille)

**Fig. 2:**
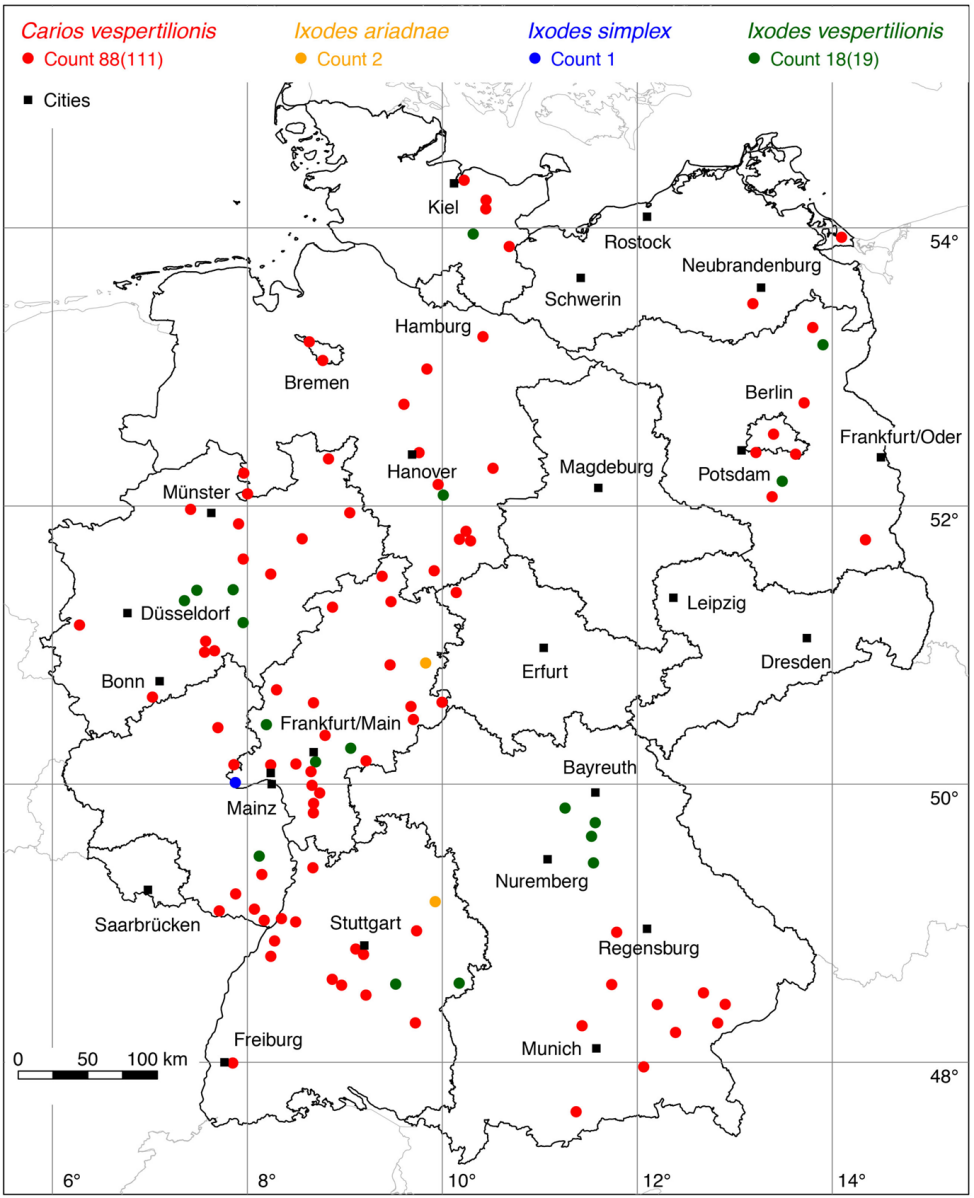
Recorded locations of *Carios vespertilionis*, *Ixodes ariadnae*, *Ixodes simplex* and *Ixodes vespertilionis* in Germany. (Color figure online)

The following 79 locations were added to the distribution map of the short-legged bat tick *C. vespertilionis* (also known as *Argas vespertilionis*): 2 (Voigts and Oudemans 1904), 2 (Schmidt 1987), 1 (Cornely and Schultz 1992), 26 (Walter 1992), 1 (Kulzer 2002), 12 (Kulzer 2003), 1 (Heddergott 2004), 1 (Hoffmeister et al. 2008), 1 (Scheffler 2011), 2 (Scheffler 2012), 1 (Scheffler 2013), 1 (Gloyna 2020), 1 (Petney 2021), 18 (legit, i.e. collected by Stefan Zaenker, 1997–2021), 9 (Rupp et al. 2004). The latter have become available to the authors through the paper by Sándor et al. (2021). A total of 88 out of 111 known locations of the soft tick *C. vespertilionis* is mapped in Fig. 2.

### *Dermacentor marginatus* (Sulzer)

**Fig. 3:**
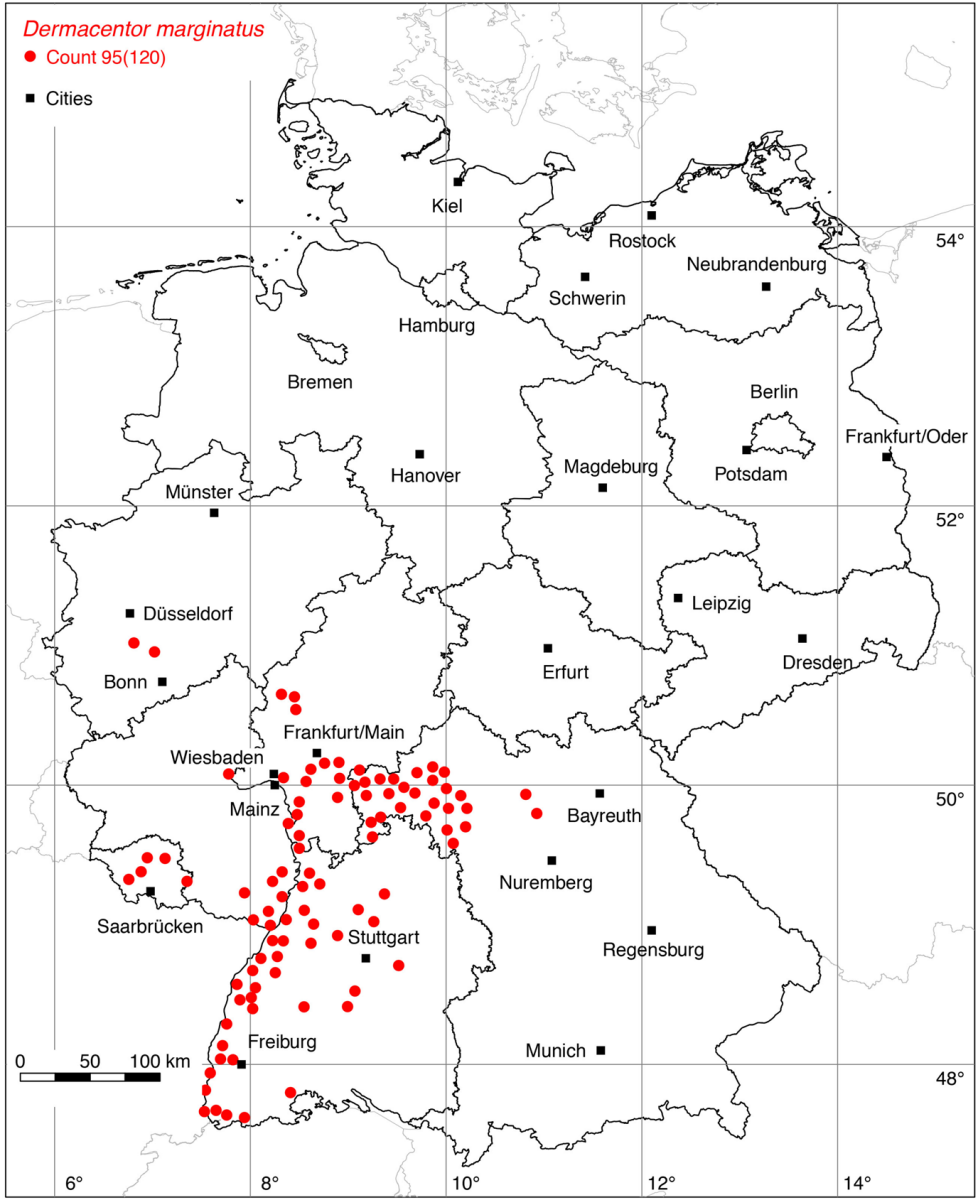
Recorded locations of *Dermacentor marginatus* in Germany. (Color figure online)

Two new locations of the ornate sheep tick *D. marginatus* were added to the distribution map. An adult male picked up by a woman south of Düsseldorf represents the new northern distribution limit of *D. marginatus* in Germany at a latitude of 51.02$^{\circ }$ N (determined by Olaf Kahl, 2021). Recent records from a citizen science study indicate that *D. marginatus* may occur even a bit further north (Springer et al. 2022). The second new tick location was reported by Weigand et al. (2023). A total of 95 out of 120 known locations is mapped in Fig. 3.

### *Dermacentor reticulatus* (Fabricius)

**Fig. 4:**
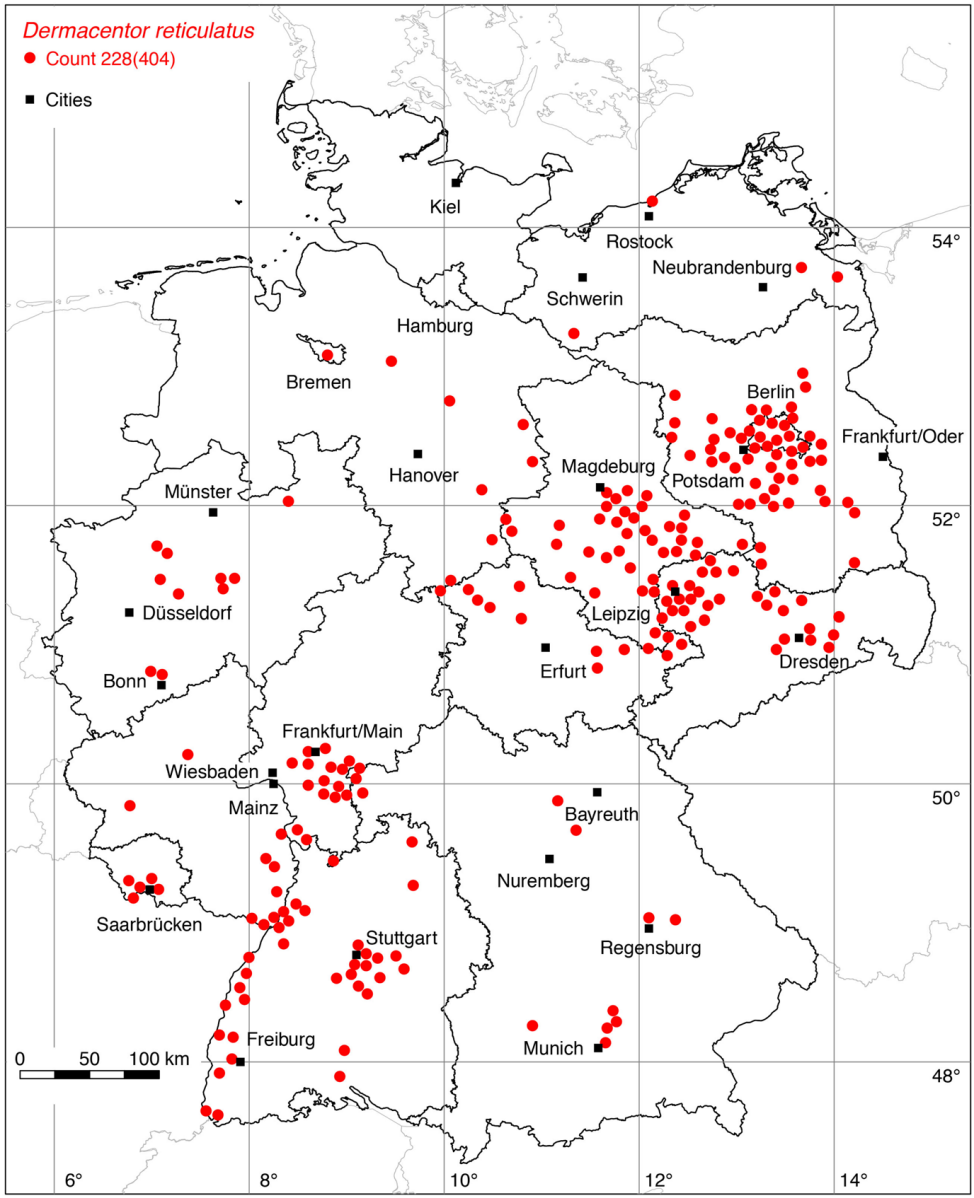
Recorded locations of *Dermacentor reticulatus* in Germany. (Color figure online)

The following 43 locations were added to the distribution map of the ornate dog tick *D. reticulatus*: 1 (Negrobov and Borodin 1964), 1 (Maasjost 2006), 19 (Liebisch and Liebisch 2007), 4 (Schreiber et al. 2014), 9 (Rehbein et al. 2016), 1 (Ott et al. 2020), 1 (leg. Olaf Kahl, 2021), 4 (Rubel et al. 2022), 3 (Răileanu et al. 2022). With the georeferenced location in Bremen (Maasjost 2006), the updated distribution map shows *D. reticulatus* findings in all federal states except Hamburg and Schleswig-Holstein. However, new records from a citizen science study were recently published for these federal states, which prove the Germany-wide occurrence of *D. reticulatus* (Springer et al. 2022). Since *D. reticulatus* has been found in Hamburg and Schleswig-Holstein but no georeferences are available, its occurrence is marked as a circle in Table 1. A total of 228 out of 404 known locations is depicted in Fig. 4.

### *Haemaphysalis (Haemaphysalis) concinna* Koch

The following four locations were added to the distribution map of *Ha. concinna*: 1 (Negrobov and Borodin 1964), 3 (Răileanu et al. 2022). A total of 22 out of 28 known locations is mapped in Fig. 4.

### *Haemaphysalis (Aboimisalis) punctata* Canestrini and Fanzago

**Fig. 5:**
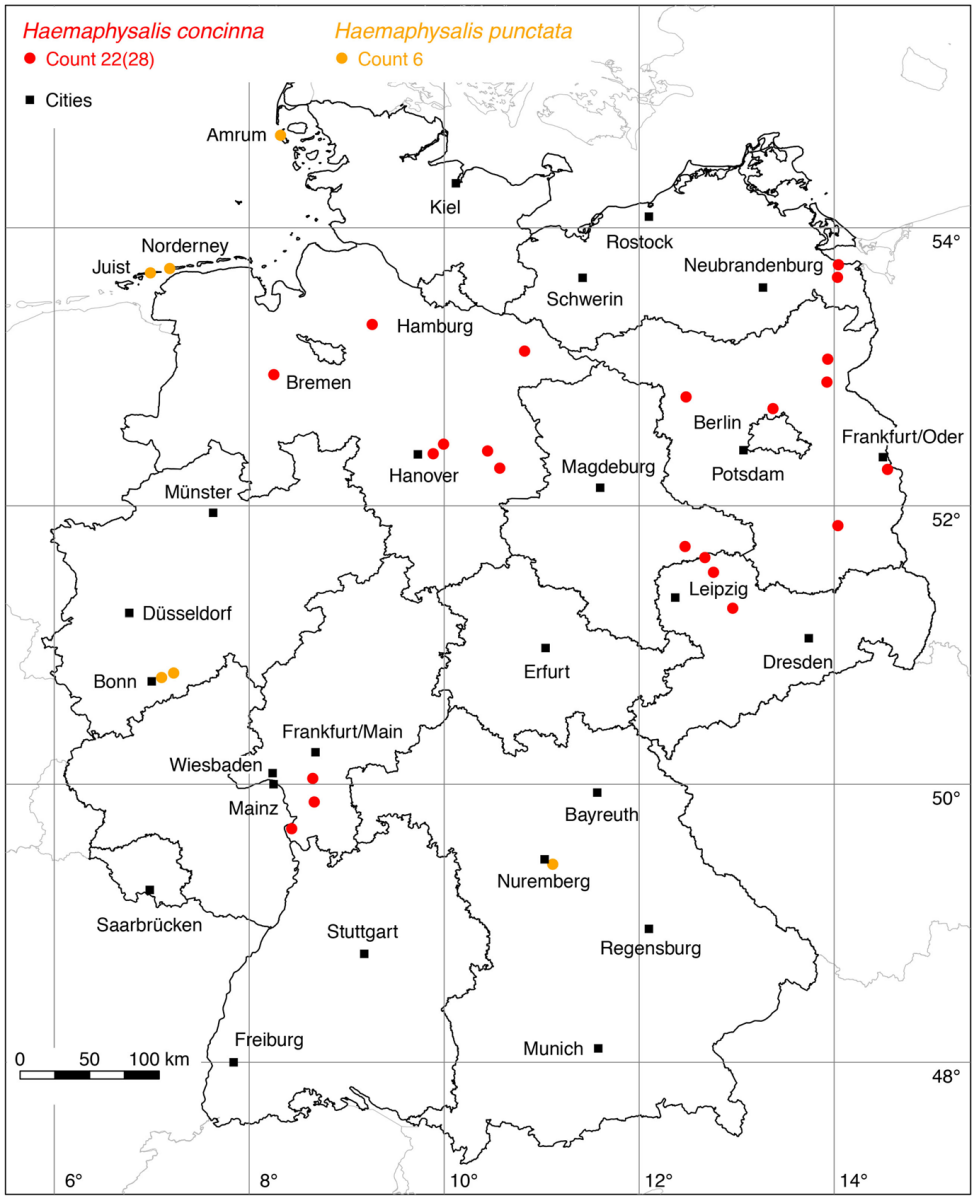
Recorded locations of *Haemaphysalis concinna* and *Haemaphysalis punctata* in Germany. (Color figure online)

The following three locations were added to the distribution map of the red sheep tick *Ha. punctata*: 1 (Koch 1877), 2 (Hesse and Völker 1983). The tick findings of Hesse and Völker (1983) were reported from Siegaue near Bonn. The meadow landscape of Siegaue is known as a resting place for migrating coastal birds, which might be why *Ha. punctata* was probably introduced by them. The ticks were collected from a stone marten (*Martes foina*) and flagged from the vegetation. The infestation of a human was also documented. The ticks found by Koch (1877) were collected near a lake at Dutzendeich, Nuremberg. The tick species described under the synonym *Rhipicephalus expositicius* is clearly *Ha. punctata* (Schulze 1925). A total of six known locations is mapped in Fig. 5.

### *Hyalomma (Euhyalomma) marginatum* Koch

**Fig. 6:**
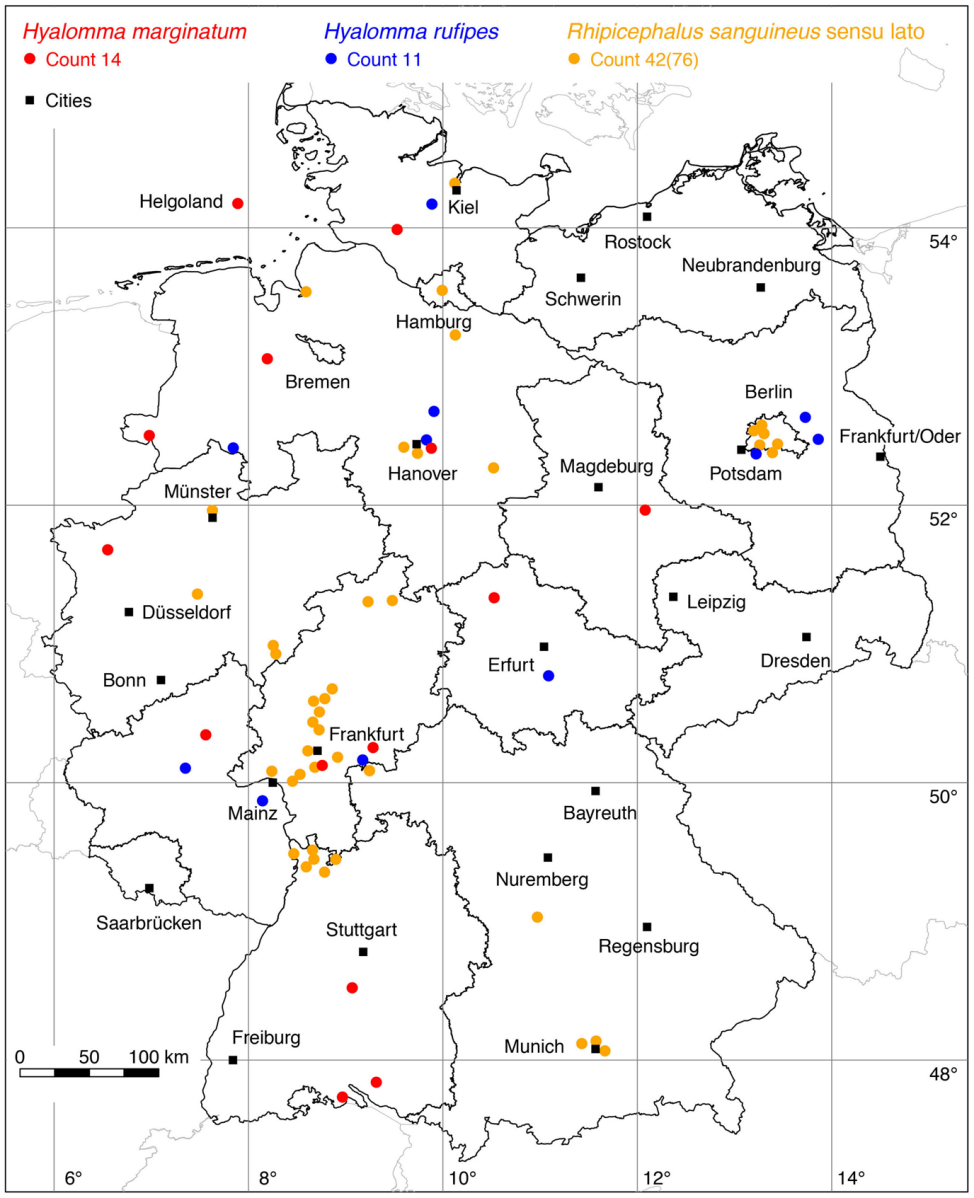
Recorded locations of *Hyalomma marginatum*, *Hyalomma rufipes* and *Rhipicephalus sanguineus* in Germany. These species are not endemic in Germany, but are continuously introduced. (Color figure online)

No locations were added to the map of *Hy. marginatum*. A total of 14 known locations is depicted in Fig. 6.

### *Hyalomma (Euhyalomma) rufipes* Koch

The following three locations were added to the map of the hairy or coarse bont-legged *Hyalomma* tick, *Hy. rufipes*: 3 (Rubel et al. 2022). A total of 11 known locations is depicted in Fig. 6.

### *Ixodes (Ixodes) acuminatus* Neumann

**Fig. 7:**
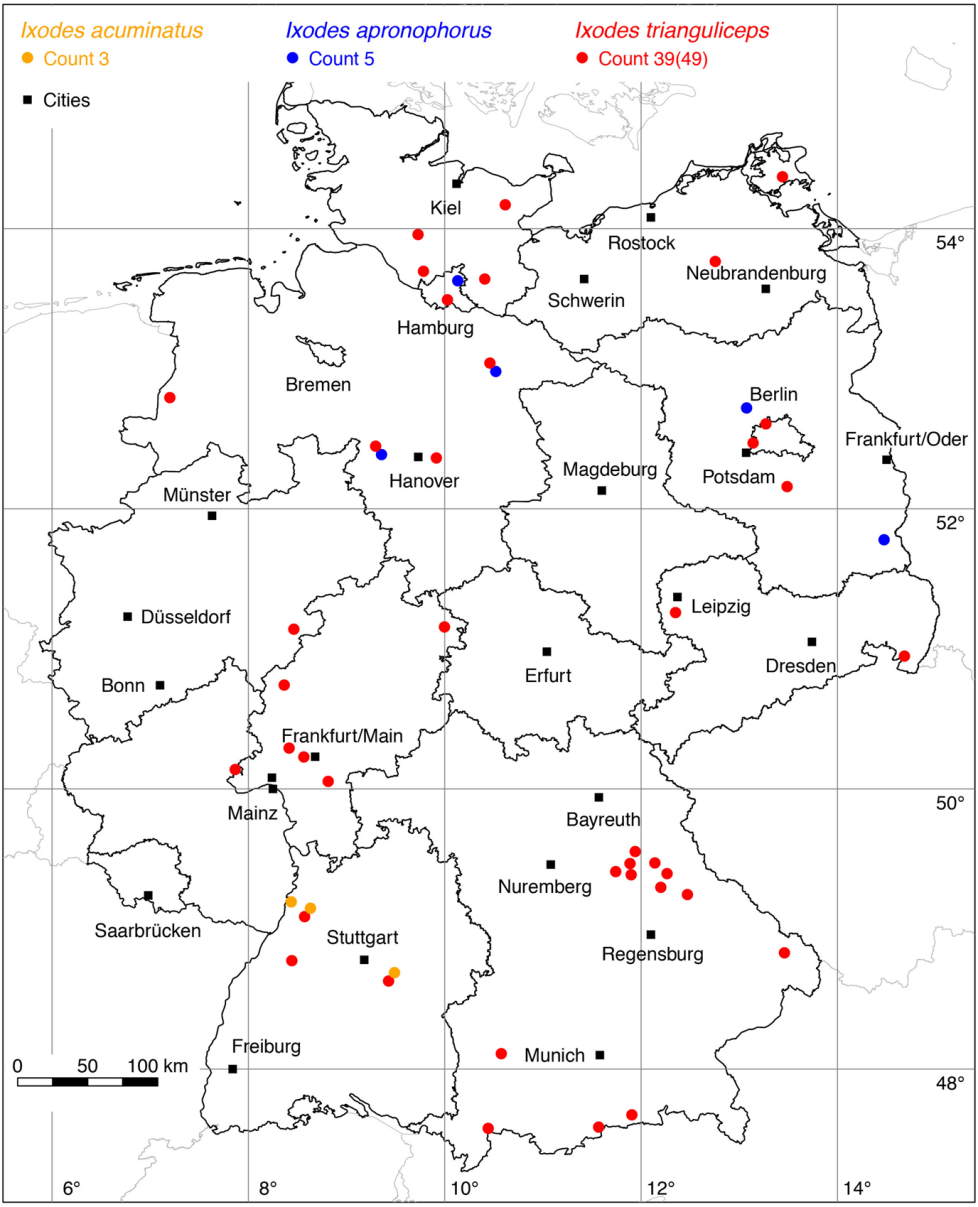
Recorded locations of *Ixodes acuminatus*, *Ixodes apronophorus* and *Ixodes trianguliceps* in Germany. (Color figure online)

No locations were added to the distribution of *I. acuminatus*. A total of three known locations is depicted in Fig. 7.

### *Ixodes (Ixodes) apronophorus* Schulze

The following three locations were added to the distribution map of *I. apronophorus*: 1 (Schulze 1924), 1 (Negrobov and Borodin 1964), 1 (Aeschlimann et al. 1970). The location of *I. apronophorus* from the first description by Schulze (1924) could be assigned to the Kremmener Luch nature reserve in Brandenburg. A total of five locations is depicted in Fig. 7.

### *Ixodes (Pholeoixodes) arboricola* Schulze and Schlottke

**Fig. 8:**
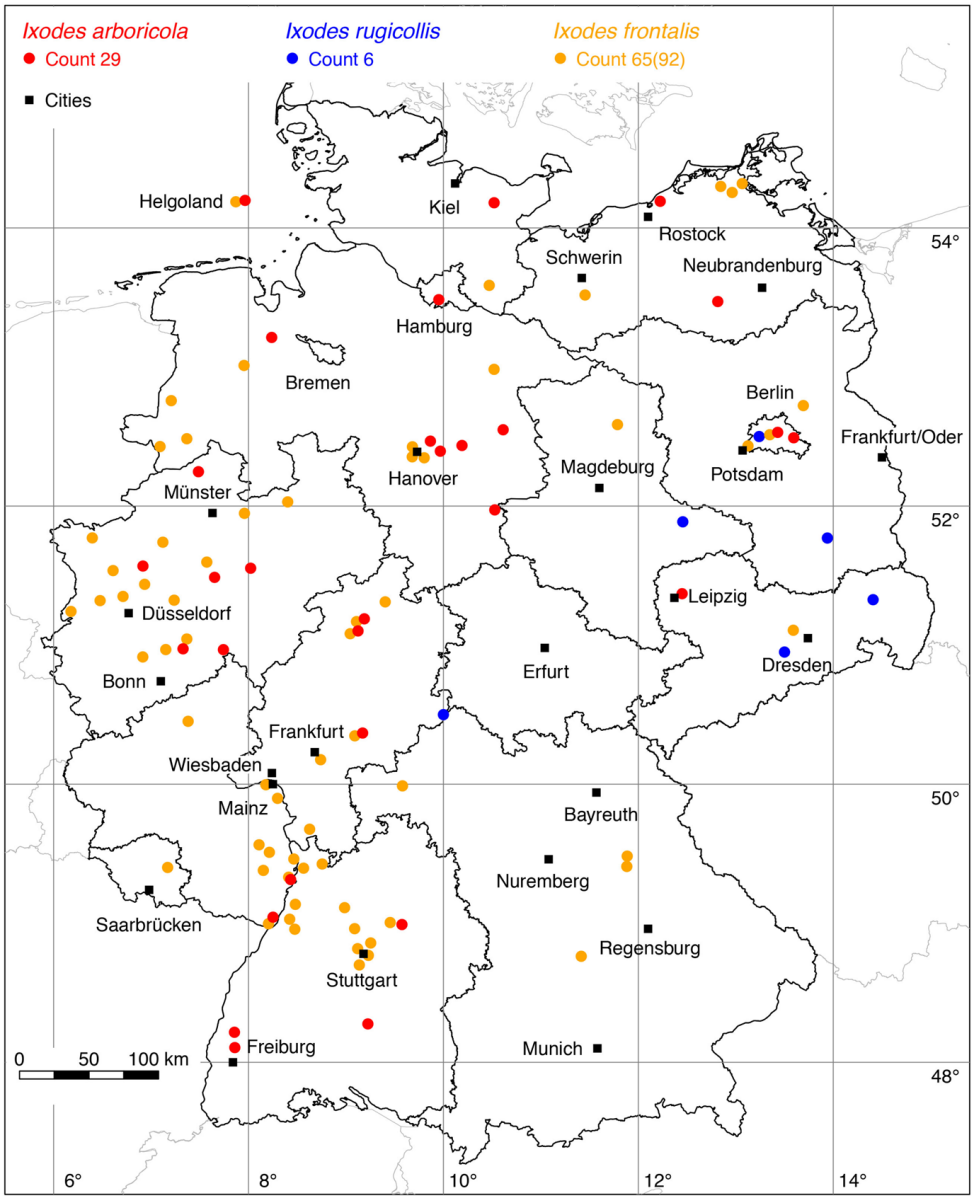
Recorded locations of *Ixodes arboricola*, *Ixodes frontalis* and *Ixodes rugicollis* in Germany. (Color figure online)

The following nine locations were added to the distribution map of *I. arboricola*: 4 (Schulze and Schlottke 1929), 1 (Schulze 1937), 1 (Schilling et al. 1981), 3 (Walter 1992). Among other places, Schulze (1932) reported *I. arboricola* from the Harz. The Harz is the highest mountain range in northern Germany (elevation: 1141 m). It lies at the intersection of Lower Saxony, Saxony-Anhalt and Thuringia. Since there are no georeferenced locations of *I. arboricola* for Saxony-Anhalt and Thuringia, its occurrence is marked as a circle in Table 1. A total of 29 known locations is depicted in Fig. 8.

### *Ixodes ariadnae* Hornok et al.

The following location was added to the distribution map of *I. ariadnae*: 1 Weigand et al. (2023). At this new location near Friedewald, Hesse, one nymph of *I. ariadnae* was found in each of the winters of 2021 and 2022. A total of two known locations is depicted in Fig. 2.

### *Ixodes (Pholeoixodes) canisuga* Johnston

**Fig. 9:**
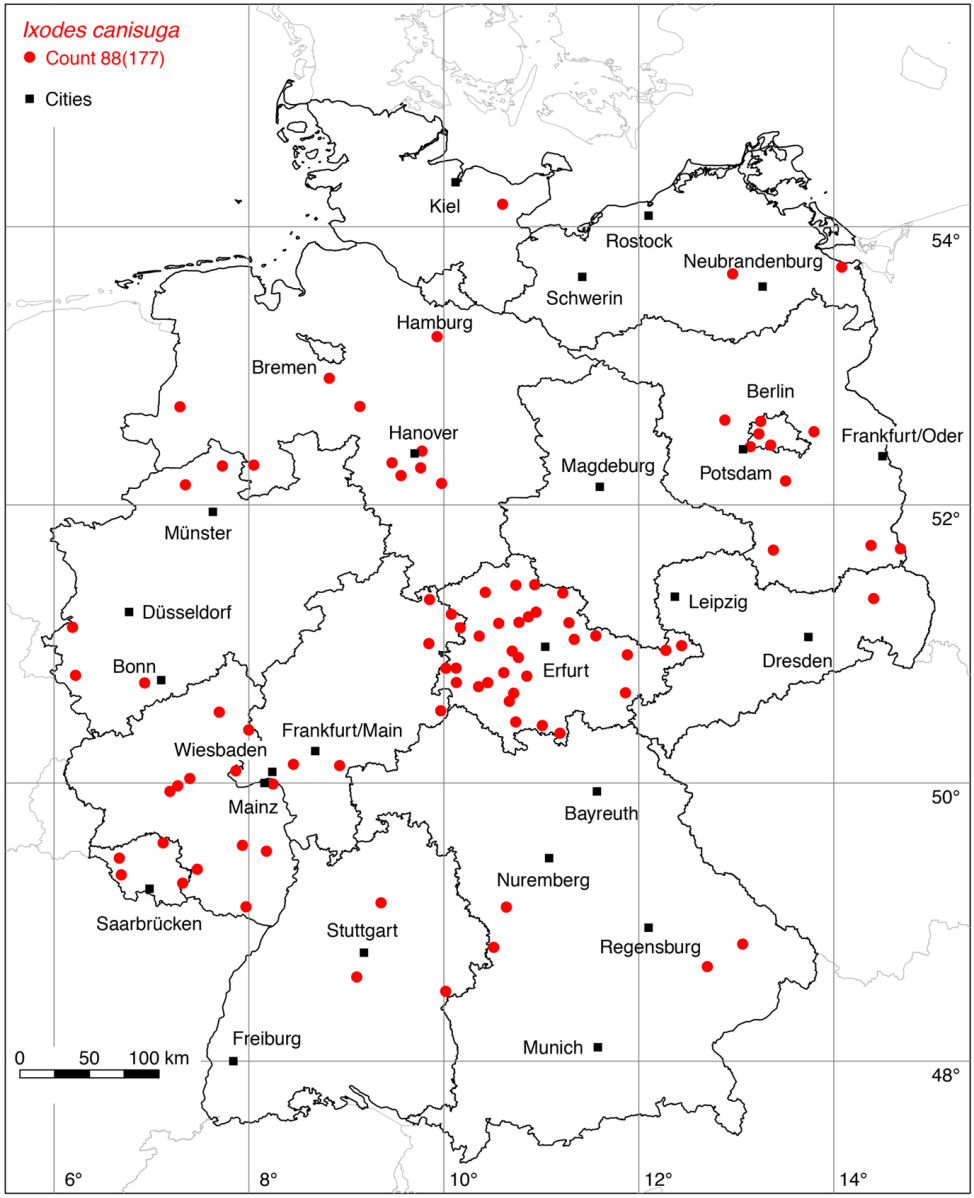
Recorded locations of *Ixodes canisuga* in Germany. (Color figure online)

The following 30 locations were added to the distribution map of the fox tick *I. canisuga*: 1 (Schulze 1918), 2 (Schulze and Schlottke 1929), 1 (Schulze 1933), 1 (Negrobov and Borodin 1964), 5 (Schöffel et al. 1991), 1 (Bröker et al. 2021), 19 Weigand et al. (2023). It should be noted that Bröker et al. (2021) originally reported a finding of *I. rugicollis* on a red fox (*Vulpes vulpes*). After a genetic redetermination by Lidia Chitimia-Dobler, this specimen was classified as *I. canisuga*. A total of 88 out of 177 known locations is depicted in Fig. 9.

### *Ixodes (Trichotoixodes) frontalis* (Panzer)

The following three locations were added to the distribution map of *I. frontalis*: 1 (Stadler and Schenkel 1940), 1 (Walter et al. 1979), 1 (Rubel et al. 2022). A total of 65 out of 92 known locations is depicted in Fig. 8.

### *Ixodes (Pholeoixodes) hexagonus* Leach

**Fig. 10:**
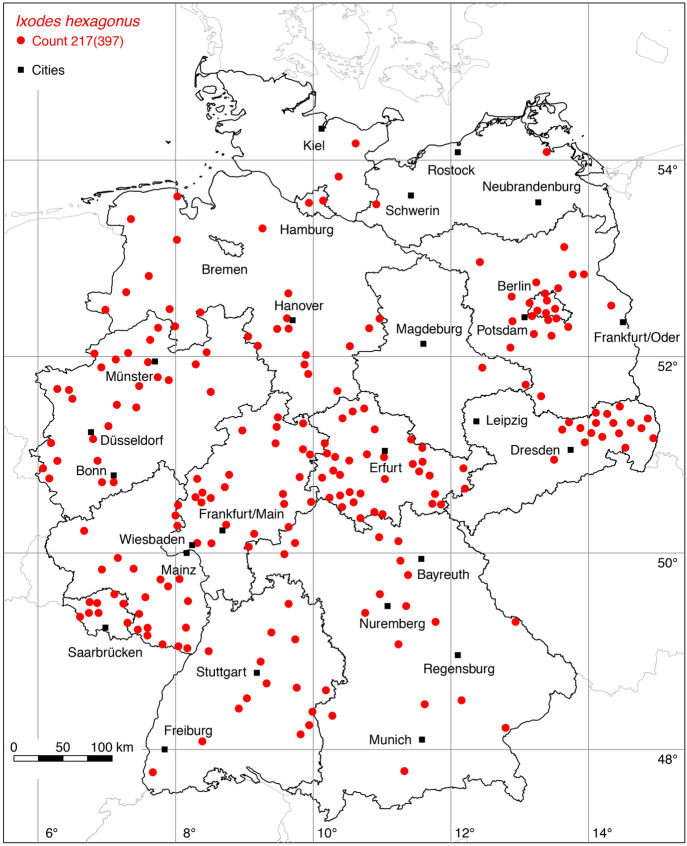
Recorded locations of *Ixodes hexagonus* in Germany. (Color figure online)

The following 80 locations were added to the distribution map of the hedgehog tick *I. hexagonus*: 2 (Schulze 1918), 1 (Schulze and Schlottke 1929), 1 (Schulze 1933), 3 (Stadler and Schenkel 1940), 1 (Negrobov and Borodin 1964), 1 (Hesse and Völker 1983), 5 (Schöffel et al. 1991), 14 (Schreiber et al. 2014), 1 (Bröker et al. 2021), 3 (Kahl et al. 2022), 2 (leg. Stefan Zaenker, 2022), 46 Weigand et al. (2023). Note that most locations depicted in the city map of Berlin (Rubel et al. 2022) are not visible in the low-resolution map of Germany presented here, although the georeferenced coordinates are provided in the supplement. A total of 217 out of 397 known locations is depicted in Fig. 10.

### *Ixodes (Ixodes) inopinatus* Estrada-Peña, Nava and Petney

New *I. inopinatus* locations in the federal state of Berlin (leg. Olaf Kahl, 2021) based on morphological identification after Estrada-Peña et al. (2014) and Chitimia-Dobler et al. (2018) have been included in Table 1. Because the majority of recent studies in Europe have not differentiated between *I. ricinus* and *I. inopinatus* and reliable differentiation of both species is very difficult, the two species are combined herein and referred to as the *I. ricinus/inopinatus* species complex. Moreover, a recent study based on genomic data indicates that German *I. inopinatus* samples may represent *I. ricinus* (Rollins et al. 2023). Consequently, it seems that the morphological and mitochondrial genome-based methods used so far are not sufficient to distinguish between *I. inopinatus* and *I. ricinus*. A separate map for *I. inopinatus* was therefore not compiled.

### *Ixodes (Pholeoixodes) lividus* Koch

The following three locations were added to the distribution map of the nest-dwelling bird parasite *I. lividus*: 2 (Schulze and Schlottke 1929), 1 (Stadler and Schenkel 1940). Müller (1977) described the occurrence of *I. lividus* in the former district of Magdeburg (former GDR). In the breeding periods 1972–1976 more than 1,800 sand martins *Riparia riparia* and some of their burrows were examined in various unspecified sand pits. The proportion of sand martins infested with *I. lividus* varied greatly from year to year between 0.7 and 6.2%. The highest infestation rate was observed in fledgling young sand martins with up to 70 larval ticks per bird. A total of eight out of nine known locations are depicted in Fig. 1.

### *Ixodes (Ixodes) ricinus* (L.)

**Fig. 11:**
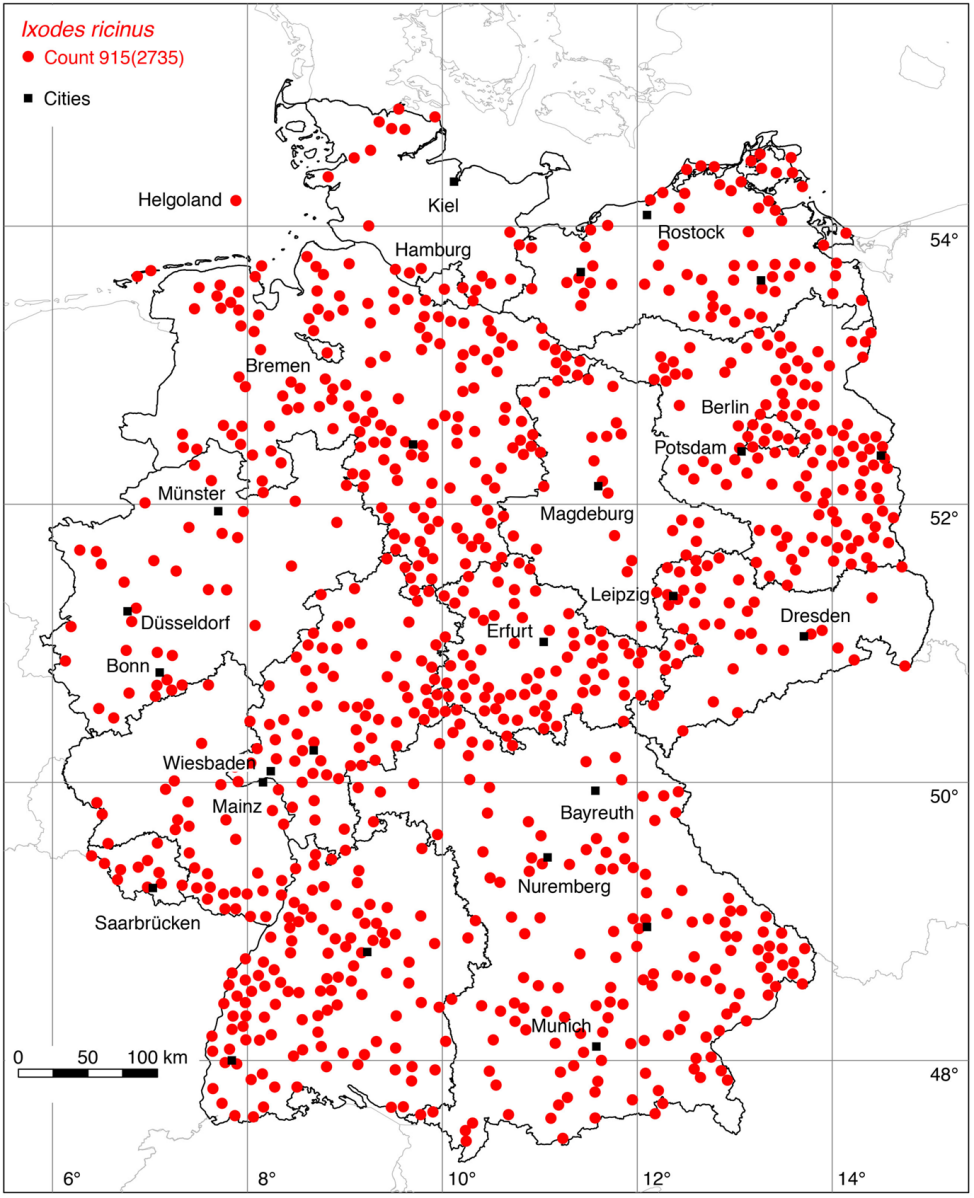
Recorded locations of the *Ixodes ricinus/inopinatus* species complex in Germany. (Color figure online)

The following 497 locations were added to the distribution map of the castor bean tick *I. ricinus*: 5 (Nuttall 1916), 2 (Stadler and Schenkel 1940), 5 (Schulze 1943), 1 (Kahmann and Halbgewachs 1962), 2 (Artz 1975), 2 (Naß 1975), 1 (Walter and Benk 1982), 1 (Henkel et al. 1983), 1 (Hesse and Völker 1983), 2 (Walter 1988), 1 (Schöffel et al. 1991), 7 (Walter 1992), 2 (Matuschka et al. 1996), 19 (Bigl et al. 1997), 4 (Fingerle et al. 1999), 1 (Kulzer 2002), 21 (Maetzel et al. 2005), 14 (Lengauer et al. 2006), 5 (Poljak 2012), 4 (Ludwig and Grosse 2014), 7 (Schreiber et al. 2014), 4 (Maaz 2018), 13 (Page et al. 2018), 15 (Müller 2019), 4 (Ott et al. 2020), 11 (Bröker et al. 2021), 21 (leg. Olaf Kahl, 2021), 5 (Răileanu et al. 2022), 32 (Rubel et al. 2022), 8 (Topp et al. 2022), 277 Weigand et al. (2023). A total of 915 out of 2,735 known locations is depicted in Fig. 11.

### *Ixodes (Pholeoixodes) rugicollis* Schulze and Schlottke

The historical Berlin location by Schulze and Schlottke (1929) was added to the distribution map of *I. rugicollis*. Since there is no exact location for this finding, the point in the map is to be interpreted symbolically for the occurrence of *I. rugicollis* in Berlin. Consequently, *I. rugicollis* was not mapped in the high-resolution city map of Berlin (Rubel et al. 2022). A total of six known locations is depicted in Fig. 8.

### *Ixodes (Pomerantzevella) simplex* Neumann

No location was added to the occurrence of *I. simplex* in Germany. The only up to now known location is depicted in Fig. 2.

### *Ixodes (Exopalpiger) trianguliceps* Birula

The following 17 locations were added to the distribution map of the shrew or vole tick *I. trianguliceps*: 2 (Schulze and Schlottke 1929), 3 (Schulze 1933), 1 (Schulze 1943), 3 (Kahmann and Halbgewachs 1962), 1 (Negrobov and Borodin 1964), 2 (Artz 1975), 1 (Walter 1981), 1 (Henkel et al. 1983), 2 (Maaz 2018), 1 (Weigand et al. 2023). A total of 39 out of 49 known locations is depicted in Fig. 7.

### *Ixodes (Ceratixodes) uriae* White

No location was added to the occurrence of the seabird tick *I. uriae* in Germany. One known location is depicted in Fig. 1.

### *Ixodes (Eschatocephalus) vespertilionis* Koch

The following 14 locations were added to the distribution map of the long-legged bat tick *I. vespertilionis*: 1 (Lengersdorf 1929), 1 (Griepenburg 1935), 1 Griepenburg (1941), 1 (Heun 1955), 1 (Negrobov and Borodin 1964), 2 (Dobat 1975), 4 (Dobat 1978), 1 (Walter and Benk 1982), 1 (Schmidt 1987), 1 (Weber 1991). The cave-dwelling tick *I. vespertilionis* was reported from the following caves: Kluterhöhle near Düsseldorf, Ahausen Höhle, Bad Segeberger Kalkberghöhle, Tunnelhöhle and Feldhofhöhle in the Hönne valley, Rosenmüllerhöhle near Muggendorf, Kollerbergloch, Petershöhle in Hartenstein, Alfelder Windloch, Hohlenstein Höhle, Gutenberger Höhle. In these caves as well as elsewhere in Germany *I. vespertilionis* parasitized the following bat species: *Rhinolophus hipposideros* (Lengersdorf 1929; Walter and Kock 1985), *Nyctalus leisleri* (Negrobov and Borodin 1964), *Myotis myotis* (Heun 1955), *Myotis mystacinus* (Walter and Benk 1982), *Myotis nattereri* (Rupp et al. 2004), *Myotis daubentonii* (Lengersdorf 1929), *Pipistrellus nathusii* (Schmidt 1987), and *Vespertilio murinus* (Griepenburg 1935). A total of 18 out of 19 known locations is depicted in Fig. 2.

### *Rhipicephalus sanguineus* (Latreille)

The following 45 locations were added to the occurrence of the brown dog tick *R. sanguineus* sensu lato: 35 (Centurier et al. 1979), 10 (Hoffmann 1981). A further study concerning 60 dogs infested with *R. sanguineus* s.l. documented the occurrence of the brown dog tick in eight federal states in West Germany (Gothe 1999). No exact location information was given in this study. However, the study documents the occurrence of *R. sanguineus* s.l. in another federal state without georeferenced locations, namely Rhineland Palatinate (Table 1). A total of 42 out of 76 known locations is mapped in Fig. 6.

## Discussion

The greatest progress compared to the first version of the atlas of ticks in Germany (Rubel et al. 2021) was made in mapping the bat ticks *C. vespertilionis* and *I. vespertilionis*. The previous atlas of ticks in Germany showed 32 locations of the short-legged bat tick *C. vespertilionis*, but these were almost all in the northwest of Germany. With another 79 locations of *C. vespertilionis* it could be shown that this tick is widespread almost all over Germany (Fig. 2). The five locations of the long-legged bat tick *I. vespertilionis* described by Walter and Kock (1985) could be supplemented by another 14 locations. Both the bats and the caves they inhabit are now under strict protection and scientific surveillance. However, there is little ongoing work on bat ticks in Germany, and ticks found in caves are only a side result of those investigations (Weigand et al. 2023). Nevertheless it can be assumed that *I. vespertilionis* is by no means rare, as the findings in numerous karst caves in the neighbouring countries Austria (Rubel and Brugger 2022) and Belgium (Obsomer et al. 2013) indicate.

With the georeferencing of 47 locations from Centurier et al. (1979), the map of *R. sanguineus* s.l. could also be significantly improved. Findings of *R. sanguineus* s.l. were reported from the metropolitan areas of Frankfurt/M., Hanover, Munich, Berlin and also from some other areas (Fig. 6). It is striking that all these findings of *R. sanguineus* s.l. are located in the former Federal Republic of Germany and in the former Berlin (West). The majority of these records date from before 1990, when people from the former East Germany were usually not allowed to visit Mediterranean or any subtropical countries. In contrast, the two *Hyalomma* species are brought to Germany via migratory birds in each spring and have been found all over the country. But because all these cases most probably reflect single (temporary) cases of importation, we do not talk about distribution. From an ongoing citizen science project (Fachet et al. 2020) 10 findings of *R. sanguineus* s.l. in Germany have been presented (not mapped), so even more current data are known. Because *R. sanguineus* s.l. in Germany usually occurs inside houses of people and quickly becomes irritating to the inhabitants, their presence might be in most cases only short-lived due to the control measures that have been introduced.

The data update also expands the knowledge of the distribution of those tick species, for which only a few new locations have been georeferenced. For example, with a new finding in Pulheim south of Düsseldorf (leg. Olaf Kahl, 2021) the northern distribution limit of *D. marginatus* in Germany shifts to the geographical latitude of 51.02$^{\circ }$ N. With the georeferencing of a location from the map of Maasjost (2006) the occurrence of *D. reticulatus* in Bremen has been documented. With updated reports of the red sheep tick *Ha. punctata* (Koch 1877; Hesse and Völker 1983) it is documented that this originally Mediterranean tick species occurs at the resting places of migratory birds on their way to Northern Europe. As a result, *Ha. punctata* is widespread on the North Sea coast of England (Tijsse-Klasen et al. 2013), the Netherlands (Hofmeester et al. 2016), and Germany (Fig. 5). The 17 newly georeferenced locations of the rarely investigated vole tick *I. trianguliceps* indicate its occurrence throughout Germany. Finally, the distribution map of the best-studied tick, *I. ricinus*, was updated to 915 plotted locations now (Fig. 11). It seems quite certain that areas without any data points of *I. ricinus* in few parts of Germany probably mirror missing investigation rather than unsuitable areas for this tick species, e.g. parts of Schleswig-Holstein in northern Germany. Mountainous areas above an altitude of 1200 m might also be an exception.

Looking at the tick data presented here from the perspective of climate change, it seems that there have been only minor effects on the German tick fauna, as yet. However, *D. reticulatus* has been found much more frequently and in much larger numbers in parts of northern Germany in the past 2–3 decades than before. It is unclear to what extent this effect is due to climate change or habitat modification. Kahl and Dautel (2013) suggested that *D. reticulatus* might profit from increasing temperatures at its northern edge of distribution because development from oviposition to the F1 adult stage must take place within only one growing season in this species. The tick *D. marginatus*, which was clearly restricted to the mild climate of the Rhine-Main area, also seems to have expanded its range somewhat to the north.

### Conclusions

The first data update of the atlas of ticks in Germany is presented here. Greatest progress compared to the first version was made in mapping the occurrence of the ticks *C. vespertilionis*, *R. sanguineus* s.l., *I. arboricola*, *I. hexagonus*, *I. trianguliceps*, and *I. vespertilionis*. The data update also expands knowledge of the distribution of rare tick species. In individual federal states, the number of documented tick species has increased by up to five. Thus, the first data update of the tick atlas in Germany and the underlying digital dataset in the supplement significantly improves our knowledge of the distribution of tick species and may be useful for future investigations to determine the effects of climate change and habitat changes on them.

## Supplementary Information

Below is the link to the electronic supplementary material.

Supplementary file1 (XLSX 100 kb)
